# The influence of gestational weight gain after bariatric procedures on selected pregnancy outcomes: a single center study

**DOI:** 10.1038/s41598-021-00549-3

**Published:** 2021-10-26

**Authors:** Maciej Walędziak, Joanna Kacperczyk-Bartnik, Paweł Bartnik, Krzysztof Czajkowski, Andrzej Kwiatkowski, Anna Różańska-Walędziak

**Affiliations:** 1 Department of General, Oncological, Metabolic and Thoracic Surgery, Military Institute of Medicine, Szaserów 128 St., Warsaw, Poland; 2 2nd Department of Obstetrics and Gynecology, Medical University of Warsaw, Karowa 2 St., Warsaw, Poland

**Keywords:** Metabolic syndrome, Obesity

## Abstract

Pregnancy after bariatric surgery is known to be associated with a higher risk of small for gestational age infants (SGA) and maternal anemia. 71 patients with a history of bariatric surgery, who had at least one pregnancy ended with a delivery of a single live-born neonate after the bariatric surgery were included in the study. The main endpoints were gestational weight gain (GWG), GWG as % of the maternal weight at the beginning of pregnancy (GWG%), maternal anemia, SGA and large for gestational age infants (LGA), neonatal intensive care unit admission (NICU). GWG% was 23.8% ± 14.1 in the LGA group vs 13.9% ± 11.0 in the normal weight neonates group; p < 0.03. Patients diagnosed with anemia before pregnancy had higher GWG% than patients without pre-pregnancy anemia (20.1% ± 11.1 vs 13.4% ± 11.6); p < 0.05. GWG% was higher in patients, whose infants were admitted to NICU (25.3% ± 17.6 vs 14.1% ± 11.0; p < 0.04). GWG% can be considered a risk predictor of the LGA and NICU admissions in bariatric patients. Anemia diagnosed before pregnancy is associated with higher GWG%.

## Introduction

Obesity has become one of the most important health issues, with an increasing prevalence worldwide. It is estimated that more than 600 million people are obese worldwide. It is associated with numerous co-morbidities, including diabetes mellitus, hypertension, obstructive sleep apnea and many others that influence the pregnancy course and neonatal outcomes. Obesity in pregnancy is related with an increased risk of gestational diabetes mellitus (GDM), pregnancy-induced-hypertension (PIH), prolonged labor and cesarean section (CS). The fetal effects of obesity include an increased incidence of congenital abnormalities, miscarriage and stillbirth^1^. While treating obesity and its adverse effects on pregnancy and neonatal outcomes, bariatric surgery can also lead to various vitamin and micronutrient deficiencies, compromising maternal and fetal well-being^2–5^. The mainstay treatment of obesity is bariatric surgery, which was proved in numerous meta-analyses to be the most effective and long-term method of treatment^6–8^. The number of bariatric procedures increases every year, with more than 2000 procedures performed in Poland in 2016 and over 600,000 worldwide^9^. Women constitute more than 80% of bariatric patients and they are mostly of reproductive age^10–12^. Pregnancy after bariatric surgery is known to be associated with a higher risk of small for gestational age infants (SGA) and maternal anemia; however the risk of large for gestational age infants (LGA), PIH and GDM is reduced^13–17^.

Gestational weight gain (GWG) is an important factor associated with perinatal outcomes, especially neonatal birth weight^18^. GWG is usually presented as the difference between maternal weight before pregnancy and the weight at the moment of delivery. In our study, we introduced GWG%, the difference between maternal weight before pregnancy and the weight at the moment of delivery presented as percentage of maternal weight before the pregnancy. The idea was similar to presenting the weight loss after bariatric operations, more commonly presented as EWL% (percent of estimated weight loss) rather than TWL (total weight loss) in kilograms, as it is more useful to evaluate the success of the operation.

### Purpose of the study

The main purpose of the study was to analyze the influence of gestational weight gain after bariatric surgery on pregnancy outcomes, the secondary aim was to investigate the possible utility of presenting the gestational weight gain as percentage of maternal weight before the pregnancy in bariatric patients.

## Results

### Basic characteristics of the study group

71 patients from the preliminary group matched the inclusion criteria of at least one single delivery of live-born neonate. The median age of the patients was 34 (31; 39) and pre-pregnancy median body mass index (BMI) 28.13 (26.42; 32.01). The median excess weight loss (EWL %) was 91.89 (76.67; 104.41), BMI at the end of pregnancy 32.46 (29.41; 37.45). The median pregnancy length was 39 (38; 40). 8 patients (11.27%) had pre-pregnancy hypertension (PPH), 12 patients (16.9%) pre-pregnancy diabetes mellitus (PGDM), 29 (40.85%) anemia. The mean time-to-conception interval after the surgery was 18 (9; 30) months.

55 patients underwent restrictive surgery (RS), 48 (67.6%) out of whom had LSG and 7 (9.85%) ASGB. 16 patients had malabsorptive surgery (MS), with 15 (21.12%) cases of LRYGB and 1 (1.4%) cases of one anastomosis gastric bypass (OAGB). The basic characteristics of patients divided into two groups is shown in Table 1.

**Table 1:** Basic characteristics of the study population.

	All
n (%)	71 (100%)
Median age, years (IQR)	34 (31; 39)
Median BMI pre-pregnancy, kg/m^2^ (IQR)	28.13 (26.4; 32.0)
Median end pregnancy BMI, kg/m^2^ (IQR)	32.46 (29.4; 37.5)
Median pregnancy length, weeks (IQR)	39 (38; 40)
Median time to conception, months (IQR)	18 (9; 30)
EWL % (IQR)	91.89 (76.7; 104.4)
PGDM, n (%)	12 (16.9%)
PPH, n (%)	8 (11.3%)
Anemia, n (%)	29 (40.8%)
GWG, kg (SD)	12.0 ± 11.2 kg
GWG%, (SD)	15.1 ± 11.7%

*BMI* body mass index, *EWL%* excess weight loss, *PGDM* pre-pregnancy diabetes mellitus, *PPH* pre-pregnancy hypertension, *SD* standard deviation, *GWG* gestational weight gain, *GWG%* gestational weight gain presented as % of pre-pregnancy maternal weight.

### General results

The mean GWG was 12.0 ± 11.2 kg GWG% was 15.1 ± 11.7%. The mean pregnancy length was 267.1 days the mean birth weight of the neonates was 3311.9 g Anemia in pregnancy was diagnosed in 29 patients (40.8%), whereas before pregnancy in 17 patients (23.9%). 12 patients had anemia both before and during pregnancy (16.9%). The incidence of GDM was 16.9% (12 patients). The incidence of PIH was 11.3% (8 patients). Pregnancy was ended with CS in 39 cases (54.9%), 61.54% out of which were performed due to elective indications, 23.08% due to suspicious of pathological cardiotocography (CTG) tracings and 15.38% due to prolonged labor. The rate of preterm deliveries was 11.3% (8 patients). 6 newborns needed NICU admission (8.5%).

### Gestational weight gain

GWG in cases with SGA was 9.8 kg ± 8.9 compared to 12.2 kg ± 11.6 in pregnancies ended with a delivery of normal birth weight neonate; p < 0.72. GWG% was 12.4% ± 10.5 in the group of neonates with SGA vs 15.6% ± 12.0% in the normal weight group; p < 0.42.

In cases with LGA GWG was 19.1 kg ± 14.9 vs 10.8 kg ± 10.2 in cases with normal birth weight; p < 0.23. The difference was statistically significant in GWG%—23.8% ± 14.1 in the LGA group vs 13.9% ± 11.0 in the normal weight group; p < 0.03.

Patients diagnosed with anemia before pregnancy had GWG 15.7 kg ± 8.3 and patients without pre-pregnancy anemia had GWG 10.8 kg ± 11.8; p < 0.06. The results presented as GWG% were of statistical significance (20.1% ± 11.1 vs 13.4% ± 11.6); p < 0.05.

Neither GWG (12.4 vs 11.5 kg), nor GWG% (15.5 vs 14.8%) differed between patients diagnosed with anemia in pregnancy and those without.

GWG (14.2 kg ± 11.6 vs 8.9 kg ± 10.30; p < 0.09) and GWG% (17.1% ± 12.5 vs 12.9% ± 10.6; p < 0.12) seemed higher in the group of pregnancies ended with CS, but these results were not of statistical significance.

GWG in pregnancies that ended with premature delivery was15.1 kg ± 12.9 vs 11.5 kg ± 11.0 in pregnancies ended at term; p < 0.59. GWG% was in pregnancies that ended with premature delivery 23.2% ± 12.8 vs 14.3% ± 11.4 in pregnancies ended at term; p < 0.08.

GWG was 21.4 kg ± 16.9 in patients whose infants were admitted to neonatal intensive care unit (NICU) compared to 11.0 kg ± 10.5 in cases without NICU admission; p < 0.23. The difference was statistically significant when presented as GWG%, with GWG% 25.3% ± 17.6 in patients whose infants were admitted to neonatal intensive care unit (NICU) vs 14.1% ± 11.0 in cases without NICU admission; p < 0.04.

GWG was 10.5 kg (± 21.0) vs 12.2 kg (± 9.5); p < 0.69 in patients who developed PIH; respectively GWG % was 14.0% (± 17.3) vs 15.2% (± 11.0); p < 0.80. In case of GDM GWG was 7.9 kg (± 6.3) vs 12.8 kg (± 11.7); p < 0.19; respectively GWG % was 9.3% (± 7.6) vs 16.2 (± 12.1); p < 0.08.

Mothers of newborns with Apgar score lower than 8 points had mean GWG of 16.0 kg (± 15.9) vs 11.6 kg (± 11.3); p < 0.43; their GWG% was 18.6% (± 18.7) vs 14.7% (± 11.7); p < 0.51.

The mean GWG was 8.9 kg (± 13.3) in patients who had TTC lower than 12 months compared to 13.8 kg (± 9.5) in those with TTC of 12 months and more; p < 0.08; respectively, GWG% was 12.6% (± 13.6) vs 16.5% (± 10.4); p < 0.19.

The relations between GWG, GWG% and pregnancy outcomes are presented in Table 2.

**Table 2:** GWG, GWG% and pregnancy outcomes.

Pregnancy outcome	GWG/GWG%	Yes	No	p value
SGA	GWG	9.8 kg ± 8.9	12.2 kg ± 11.6	< 0.72
	GWG%	12.4% ± 10.5	15.6% ± 12.0%	< 0.42
LGA	GWG	19.1 kg ± 14.9	10.8 kg ± 10.2	< 0.23
	GWG%	23.8% ± 14.1	13.9% ± 11.0	< 0.03
Anemia before pregnancy	GWG	15.7 kg ± 8.3	10.8 kg ± 11.8	< 0.06
	GWG%	20.1% ± 11.1	13.4% ± 11.6	< 0.05
Anemia during pregnancy	GWG	12.4 ± 9.5	11.5 ± 12.3	< 0.81
	GWG%	12.4 ± 10.5%	15.6% ± 12.0	< 0.42
CS	GWG	14.2 kg ± 11.6	8.9 kg ± 10.30	< 0.09
	GWG%	17.1% ± 12.5	12.9% ± 10.6	< 0.12
Premature delivery	GWG	15.1 kg ± 12.9	11.5 kg ± 11.0	< 0.59
	GWG%	23.2% ± 12.8	14.3% ± 11.4	< 0.08
NICU admission	GWG	21.4 kg ± 16.9	11.0 kg ± 10.5	< 0.23
	GWG%	25.3% ± 17.6	14.1% ± 11.0	< 0.04
PIH	GWG	10.5 kg ± 21.0	12.2 kg ± 9.5	< 0.69
	GWG%	14.0% ± 17.3	15.2% ± 11.0	< 0.80
GDM	GWG	7.9 kg ± 6.3	12.8 kg ± 11.7	< 0.19
	GWG%	9.3% ± 7.6	16.2% ± 12.1	< 0.08
Apgar score < 8	GWG	16.0 kg ± 15.9	11.6 kg ± 11.3	< 0.43
	GWG%	18.6% ± 18.7	14.7% ± 11.7	< 0.51
TTC < 12 months	GWG	8.9 kg ± 13.3	13.8 kg ± 9.5	< 0.08
	GWG%	12.6% ± 13.6	16.5% ± 10.4	< 0.19

*SGA* small for gestational age infants, *LGA* large for gestational age infants, *CS* cesarean section, *NICU* neonatal intensive care unit, *PIH* pregnancy induced hypertension, *GDM* gestational diabetes mellitus, *TTC* time from surgery to conception.

We did not find statistical significance between SGA, LGA, pregnancy anemia, pre-pregnancy anemia, cesarean section, preterm delivery or NICU admission of the newborn and time-to-conception interval. The relations between pregnancy complications and perinatal outcomes with time from surgery to conception (TTC) are presented in Table 3.

**Table 3:** Time-to-conception in months depending on the perinatal outcomes.

1-present 0-absent		Time to conception in months	p-value
SGA	1	24.7 ± 14.2	< 0.50
	0	21.0 ± 17.3	
LGA	1	24.2 ± 20.9	< 0.61
	0	21.2 ± 16.3	
Pre-pregnancy anemia	1	27.0 ± 23.5	< 0.12
	0	19.7 ± 13.9	
Pregnancy anemia	1	20.0 ± 15.4	< 0.53
	0	22.6 ± 17.9	
Cesarean section	1	22.4 ± 19.2	< 0.72
	0	20.9 ± 13.7	
Preterm delivery	1	28.0 ± 28.8	< 0.29
	0	20.8 ± 15.1	
NICU	1	21.0 ± 14.5	< 0.95
	0	21.5 ± 17.2	

*SGA* small for gestational age, *LGA* large for gestational age, *NICU* neonatal intensive care unit.

## Discussion

An innovation of our study is presenting GWG as GWG% (% of the maternal weight at the beginning of pregnancy). GWG% seems to be a better predictor of adverse pregnancy and neonatal outcomes than GWG, as it presents more accurately the real proportion of maternal weight gain during pregnancy. One of the principal findings of our study was that GWG presented as % of the maternal weight at the beginning of pregnancy could be a predictor of the LGA risk in bariatric patients. Even though there is a decrease in proportion of LGA in pregnancies after bariatric surgery, it still remains an important problem for the obstetricians, especially when the patient remains to be obese after the surgery. We found mean GWG% of almost 24% in pregnancies ended with a delivery of LGA, compared to 14% in cases with normal birth weight of the neonates; p < 0.03. GWG% was higher in pregnancies ended with NICU admission of the neonate (25 vs 14%); p < 0.04.

The impact of gestational weight gain on perinatal outcome was analyzed in a recent French study^18^. The researchers investigated 337 pregnancies in 264 women after bariatric surgery, They evaluated GWG and found that insufficient GWG compared to excessive GWG was associated with an increased rate of SGA and preterm labor. In our study, GWG was 9.8 vs 12.2 kg and GWG% 12.4 vs 15.6% in cases with SGA than in pregnancies ended with a delivery of normal birth weight neonate, although our results were not of statistical significance and therefore cannot be interpreted as findings of our study.

Rottenstreich et al. published a systematic review of 27 articles about maternal nutritional status and related pregnancy outcomes following bariatric surgery^3^. They analyzed the influence of micronutrient and vitamin deficiencies typical for post-operative bariatric patients. Some of the deficiencies, such as iron, folate, vitamins B1, B12 and D occurred both after restrictive and malabsorptive procedures. Additionally, they found an increased risk of fetal malnutrition and impaired intrauterine growth if the pregnancy started in the rapid catabolic weight loss period after the operation. They found a correlation between a higher rate of maternal anemia and a longer TTC. According to the authors, this may be partially explained by a lower adherence to nutritional supplementation post-operative recommendations correlated with the time lapsed from surgery. The positive correlation between TTC and maternal anemia during pregnancy is also confirmed in other studies^19,20^.

In our study the mean TTC was 27.0 in patients who were diagnosed with anemia before pregnancy vs 19.7 months in those who were not diagnosed with anemia before pregnancy. The mean TTC in patients with pregnancy anemia was 20.0 vs 22.6 months. However, these results did not present statistical significance. The incidence of anemia in our study group was higher during than before pregnancy (26 vs 19%). Patients diagnosed with anemia before pregnancy had higher GWG% than patients without anemia (20 vs13%); p < 0.05.

The impact of maternal anemia after bariatric surgery on birth weight is discussed in many studies. Coupaye et al. analyzed a group of 123 patients who had undergone bariatric procedures before pregnancy and found a negative correlation between the maternal iron serum level and neonatal birth weight^4^.

A Swedish national cohort study investigated pregnancy and neonatal outcomes in women after bariatric surgery and found a higher risk of SGA (15.6 vs 7.6%) and an association between a longer TTC and a higher risk of SGA^21^, the correlation confirmed also in other studies^13,22,23^.

Bariatric surgery is associated with a higher incidence of IUGR and SGA, therefore pregnancies after bariatric surgery should be considered high-risk and closely monitored by a multidisciplinary team^11,24^. Maternal anemia in pregnancy after bariatric surgery is an important problem that may impair fetal growth and result in necessity of blood transfusion and longer hospitalization after the delivery. The risk of anemia increases with the time from surgery to conception. Patients supplementation adherence should be monitored in the time of periconception.

Gestational weight gain after bariatric surgery may be an independent factor associated with pregnancy and neonatal outcomes. GWG% seems to be a better predictor of adverse outcomes than GWG, as it presents more accurately the real proportion of maternal weight gain during pregnancy. LGA, though less common after bariatric operations still remains an important obstetric issue and according to our study, higher GWG% is positively related with LGA. GWG% is also positively associated with anemia before pregnancy and NICU admission of the neonate. More correlations between GWG% and adverse pregnancy and neonatal outcomes are to be further investigated.

## Methods

This study was designed as an online and paper survey with the aim to collect data about gynecological and obstetric characteristics of Polish women after bariatric operations. We collected data from 107 female patients with a history of bariatric surgery who had a history of at least one pregnancy after bariatric surgery. 74 patients had laparoscopic sleeve gastrectomy (LSG), 21 laparoscopic Roux-en-Y gastric banding (LRYGB), 9 adjustable silicone gastric banding (ASGB) and 3 other bariatric procedures. 71 patients met the inclusion criteria—having had at least one pregnancy ended with a delivery of a single live-born neonate after the bariatric surgery. The exclusion criteria were multiple pregnancies, stillbirth, exclusive miscarriages and no sufficient data for the analysis. The primary group were 102 women, 22 out of whom were excluded as their pregnancies ended only with miscarriages, 3 had twin pregnancies and in 7 cases there was no sufficient data. We considered stillbirth as an exclusion criterium, but no patients in our group had stillbirth. The vast majority of patients had spontaneous pregnancies, only one patient reported having become pregnant after using methods of reproductive assistance. All patients included in the study received oral vitamin and micronutrient supplementation throughout the pregnancy, according to international recommendations^10^. The endpoints were gestational weight gain (GWG), GWG presented as percentage of pre-pregnancy maternal weight (GWG%). GDM, PIH, anemia before and during pregnancy, pregnancy length, premature deliveries, birth weight of the neonate, small for gestational age infants (SGA), large for gestational age infants (LGA) and way of delivery.

GWG was defined as the difference between maternal weight before pregnancy and the weight on the day of delivery or the nearest day to the delivery possible, presented in kilograms. GWG% was defined as the difference between maternal weight before pregnancy and the weight at the moment of delivery as percentage of maternal weight before the pregnancy.

Although our national recommendations follow the international guidelines about diagnosing GDM in patients after bariatric surgery and the lack of effectiveness and appropriateness of oral glucose tolerance test (OGTT), the vast majority of patients admitted having had OGTT with 75 g glucose administered by their obstetricians between 24 and 28th week of gestation. “*GDM was identified in case of fasting blood glucose level of* ≥ *5.1 mmol/l up to 6.9 mmol/l,* ≥ *10 mmol/l after first hour of glucose administration and/or* ≥ *8.5 mmol/l up to 11.0 mmol/l after the second hour. PIH was defined as *de novo* onset of hypertension (*> *140 mmHg systolic or* > *90 mmHg diastolic) after 20*^*th*^* week of gestation”,* as defined in one of our previous studies^30^*.*

Anemia before pregnancy was defined as hemoglobin plasma concentration below 12 g/l diagnosed before the pregnancy. Anemia in pregnancy was diagnosed when hemoglobin plasma concentration decreased below 11 g/dl in first and third trimester and 10.5 g/dl in second trimester.

“SGA was defined as below the 10th percentile and LGA as over 90th percentile using population adjusted birth weight scores”^30^.

EWL% was the excess weight loss, interpreted as the proportion of the optimum weight loss for the patient, based on the preoperative weight and optimum weight.

NICU was defined as the necessity of admission of a newborn to the Neonatal Intensive Care Unit at any time of hospitalization.

We considered TTC of 12 months or more an acceptable time interval.

### Statistical analysis

Statistical analysis was performed using Statistica 13 (StatSoft. Inc.). U‐Mann Whitney test and t‐student tests were used for single-dimension quantitative data comparison as required. Two‐sided Fisher's exact test was used for categorical and binary data comparison. Due to group size (n = 71) no multi-dimensional analysis was performed p value < 0.05 was considered significant.

### Ethical considerations

The study was anonymous, performed in accordance with the ethical standards aid down in the 1964 Declaration of Helsinki and its latter amendments (Fortaleza). Participants were informed about the aim of the study and informed consent was obtained electronically prior to the beginning of the survey. The approval from Military Institute of Medicine Ethics Committee was obtained 22–08-2018 from code 117/WIM/2018.

### Limitations of the study

The main limitation of our study is the possibility of recall bias giving the retrospective nature of the study. The other limitation is the sample size, especially in the group of malabsorptive surgery. Additionally, due to group size (n = 71) no multi-dimensional analysis was performed.

### Consent to participate

Participants were informed about the aim of the study and informed consent was obtained electronically prior to the beginning of the survey.

## Data Availability

Correspondence and requests for materials should be addressed to M.W.

## Abbreviations

GDM: Gestational diabetes mellitus
PIH: Pregnancy induced hypertension
CS: Cesarean section
SGA: Small for gestational age infants
CHLO: Polish Association of Bariatric Patients
LSG: Laparoscopic sleeve gastrectomy
LRYGB: Laparoscopic Roux-en-Y gastric banding
ASGB: Adjustable silicone gastric banding
GWG: Gestational weight gain
LGA: Large for gestational age infants
OGTT: Oral glucose tolerance test
BMI: Body mass index
EWL%: Excess weight loss
PPH: Pre-pregnancy hypertension
PGDM: Pre-pregnancy diabetes mellitus
RS: Restrictive surgery
MS: Malabsorptive surgery
GWG%: Gestational weight gain presented as % of pre-pregnancy maternal weight
CTG: Cardiotocography
NICU: Neonatal intensive care unit
TTC: Time from surgery to conception
IUGR: Intrauterine growth restriction

## References

[1] ACOG Practice Bulletin (2009). Clinical management guidelines for obstetrician-gynecologists. Obstet Gynecol..

[2] Slater C, Morris L, Ellison J (2017). Nutrition in pregnancy following bariatric surgery. Nutrients.

[3] Rottenstreich A, Elazary R, Goldenshluger A (2019). Maternal nutritional status and related pregnancy outcomes following bariatric surgery: A systematic review. Surg. Obes. Relat. Dis..

[4] Coupaye M, Legardeur H, Sami O (2018). Impact of Roux-en-Y gastric bypass and sleeve gastrectomy on fetal growth and relationship with maternal nutritional status. Surg. Obes. Relat. Dis..

[5] Yau PO, Parikh M, Saunders JK (2017). Pregnancy after bariatric surgery: The effect of time-to-conception on pregnancy outcomes. Surg. Obes. Relat. Dis..

[6] Neylan CJ, Kannan U, Dempsey DT (2016). The surgical management of obesity. Gastroenterol. Clin. N. Am..

[7] Lindekilde N, Gladstone BP, Lübeck M (2015). The impact of bariatric surgery on quality of life: A systematic review and meta-analysis. Obes. Rev..

[8] Gloy VL, Briel M, Bhatt DL (2013). Bariatric surgery versus non-surgical treatment for obesity: A systematic review and meta-analysis of randomised controlled trials. BMJ.

[9] Walędziak M, Różańska-Walędziak A, Kowalewski PK (2019). Present trends in bariatric surgery in Poland. Wideochir Inne Tech Maloinwazyjne..

[10] Shawe J, Ceulemans D, Akhter Z (2019). Pregnancy after bariatric surgery: Consensus recommendations for periconception, antenatal and postnatal care. Obes. Rev..

[11] Rottenstreich A, Elazary R, Levin G (2018). Pregnancy after bariatric surgery and the risk of fetal growth restriction. Surg. Obes. Relat. Dis..

[12] Carreau A-M, Nadeau M, Marceau S (2017). Pregnancy after bariatric surgery: Balancing risks and benefits. Can. J. Diabetes..

[13] Akhter Z, Rankin J, Ceulemans D (2019). Pregnancy after bariatric surgery and adverse perinatal outcomes: A systematic review and meta-analysis. PLoS Med..

[14] Watanabe A, Seki Y, Haruta H (2019). Maternal impacts and perinatal outcomes after three types of bariatric surgery at a single institution. Arch. Gynecol. Obstet..

[15] Haseeb YA (2019). A review of obstetrical outcomes and complications in pregnant women after bariatric surgery. Sultan Qaboos Univ. Med. J..

[16] Malik S, Teh JL, Lomanto D (2020). Maternal and fetal outcomes of Asian pregnancies after bariatric surgery. Surg. Obes. Relat. Dis..

[17] Kominiarek MA (2011). Preparing for and managing a pregnancy after bariatric surgery. Sem. Perinatol..

[18] Grandfils S, Demondion D, Kyheng M (2019). Impact of gestational weight gain on perinatal outcomes after a bariatric surgery. J. Gynecol. Obstet. Hum. Reprod..

[19] Falcone V, Stopp T, Feichtinger M, Kiss H (2018). Pregnancy after bariatric surgery: A narrative literature review and discussion of impact on pregnancy management and outcome. BMC Pregnancy Childbirth.

[20] Nomura RMY, Dias MCG, Igai AMK, Paiva LV, Zugaib M (2011). Anemia during pregnancy after silastic ring Roux-en-Y gastric bypass: Influence of time to conception. Obes. Surg..

[21] Johansson K, Cnattingius S, Naslund I (2015). Outcomes of pregnancy after bariatric surgery. NEJM.

[22] Chevrot A, Kayem G, Coupaye M, Lesage N, Msika S, Mandelbrot L (2016). Impact of bariatric surgery on fetal growth restriction: Experience of a perinatal and bariatric surgery center. Am. J. Obstet. Gynecol..

[23] Rottenstreich A, Elchalal U, Kleinstern G (2018). Maternal and perinatal outcomes after laparoscopic sleeve gastrectomy. Obstet. Gynecol..

[24] Różańska-Walędziak A, Walędziak M, Bartnik P (2020). The influence of bariatric surgery on pregnancy and perinatal outcomes—A case-control study. J. Clin. Med..

